# Identifying the Subtypes of Major Depressive Disorder Based on Somatic Symptoms: A Longitudinal Study Using Latent Profile Analysis

**DOI:** 10.3389/fpsyt.2022.759334

**Published:** 2022-07-12

**Authors:** Xiaohui Wu, Yuncheng Zhu, Zhiguo Wu, Jia Huang, Lan Cao, Yun Wang, Yousong Su, Hongmei Liu, Maosheng Fang, Zhijian Yao, Zuowei Wang, Fan Wang, Yong Wang, Daihui Peng, Jun Chen, Yiru Fang

**Affiliations:** ^1^Clinical Research Center and Division of Mood Disorders, Shanghai Mental Health Center, Shanghai Jiao Tong University School of Medicine, Shanghai, China; ^2^Wuhan Mental Health Centre, Wuhan, China; ^3^Nanjing Medical University Affiliated Brain Hospital, Nanjing, China; ^4^Department of Psychiatry, Hongkou District Mental Health Center of Shanghai, Shanghai, China; ^5^CAS Center for Excellence in Brain Science and Intelligence Technology, Shanghai, China; ^6^Shanghai Key Laboratory of Psychotic Disorders, Shanghai, China

**Keywords:** major depression disorder, somatic symptom, latent profile analysis, subtype, GLMM

## Abstract

**Background:**

Two-thirds of major depressive disorder (MDD) patients initially present with somatic symptoms, yet no study has used approaches based on somatic symptoms to subtype MDD. This study aimed to classify MDD *via* somatic symptoms and tracked the prognosis of each subtype.

**Methods:**

Data were obtained from the study of Algorithm Guided Treatment Strategies for Major Depressive Disorder (AGTs-MDD). We recruited 395 subjects who received monotherapy of mirtazapine or escitalopram and conducted 2-, 4-, 6-, 8-, and 12-week follow-up assessments (*n* = 311, 278, 251, 199, and 178, respectively). Latent profile analysis (LPA) was performed on somatic symptom items of the depression and somatic symptoms scale (DSSS). Generalized linear mixed models (GLMM) were used to study the longitudinal prognosis of the subtypes classed by LPA. Primary outcome measures were the Hamilton Depression Rating Scale (HAMD), HAMD score reduction rate, as well as somatic and depressive items of DSSS.

**Results:**

Three subtypes of MDD were found, namely, depression with mild somatic symptoms (68.9%), depression with moderate somatic symptoms (19.2%), and depression with severe somatic symptoms (11.9%). Scores of HAMD (*F* = 3.175, *p* = 0.001), somatic (*F* = 23.594, *p* < 0.001), and depressive (*F* = 4.163, *p* < 0.001) DSSS items throughout the 12-week follow-up showed statistical difference among the three subtypes. The moderate group displayed a higher HAMD-17 score and a lower reduction rate at the 6th week, and more severe depressive symptoms both at the 4th and 6th weeks.

**Conclusion:**

The results indicate that somatic symptoms should be emphasized in patients with MDD, and more attention is needed for those with moderate somatic symptoms, which may be relevant to a worse prognosis.

## Introduction

For all ages and both sexes combined, the prevalence of major depressive disorder (MDD) was approximately 2.21% globally in 2017 (1). A nationwide cross-sectional epidemiological survey across China showed that the lifetime prevalence of MDD is around 3.36% (2). A multicenter international study reported that two-thirds of MDD patients initially present with somatic symptoms (3). Moreover, patients with somatic symptoms tended to co-exist with both depressive and anxiety disorders. It has been reported that depressed patients are 4.43 times more likely to have somatoform disorders than non-depressed ones (4). Indeed, Chinese respondents were more likely to complain about somatic symptoms rather than psychological symptoms in comparison with patients in western countries. Due to the influence of culture, over 50% of Chinese MDD patients with somatic symptoms first seek for medical consultation (5, 6). Previous evidence proved the strong associations between depression and somatic symptoms; however, most research has focused only on depression (7).

Somatic symptoms of MDD can be grouped into (1) vegetative symptoms, including sleep disturbance, changes in appetite, and lack of energy; (2) painful symptoms, including headache, backache, gastrointestinal disturbances, and musculoskeletal aches; and (3) non-painful symptoms, including dizziness, palpitations, dyspnea, and shortness of breath (8, 9). Neurovegetative symptoms are included in the most core symptoms of depression (10). Complaints of multiple pain in patients with MDD were reported to be positively related to severe emotional symptoms (11). Besides, the symptom of pain may worsen the treatment response of depression, and this residual symptom could largely increase the disease burden (12). Previous studies have demonstrated that somatic symptoms were predictors for greater severity, worse prognosis, and poorer treatment response, as well as the chronicity and delayed remission of MDD. It has been reported that the cardiopulmonary, gastrointestinal, and general symptomatic cluster could predict the 2-year persistence of MDD. The presence of multiple somatic symptoms was a significant predictor (OR = 1.69, 95%CI = 1.07–2.68, *p* = 0.03) (13–15). Even after appropriate treatment, somatic symptoms may remain as residual symptoms, hindering the remission and increasing the risk of relapse (16).

In the *Diagnostic and Statistical Manual of Mental Disorders, Fifth Edition* (DSM-5), MDD is classified into 13 subtypes by clinical features. However, MDD is a heterogeneous syndrome, with which patients differ remarkably in symptoms, treatment responses, and pathophysiological mechanisms. In clinical practice, patients with MDD often show opposite profiles of symptoms, such as increase or decrease in appetite, hyposomnia, or hypersomnia. Therefore, DSM-5 diagnostic classifications may not be specific enough to generalize sophisticated phenotypes of MDD. Over the decades, researchers have been trying to categorize depression into different subgroups. In general, previous studies have already tried to classify depression based on clinical symptoms, medication responses, neuroimaging, genetics, and neurotransmitter distributions (17–20). To the best of our knowledge, somatic symptoms have been neglected or haven't been mainly considered when subdividing MDD.

In clinical practice, diagnosis of MDD mainly depends on emotional symptoms rather than somatic symptoms, which may be influenced by different expressions of depressive symptoms, especially in China, where people tend to express their somatic symptoms rather than emotional problems (9). The Depression and Somatic Symptoms Scale (DSSS) is a reliable questionnaire, which can assess and monitor the severity of both depressive and somatic symptoms. DSSS is composed of two major subscales, namely, the depressive subscale (DS), including 12 items, and the somatic subscale (SS), including 10 items (21). The DS, SS, and Hamilton Depression Rating Scale (HAMD) scores at baseline were reported to be significantly associated with the long-term outcome of depression. Besides, the scales or subscales for assessing somatic symptoms might be more strongly associated with the outcome of depression (22). The total score of DSSS ranges from 0 to 66, in which DS ranges from 0 to 36 and SS ranges from 0 to 30. The items of SS are designed to reflect the common somatic symptoms of MDD, which can reflect the severity of depression and have a significant impact on the prognosis of patients (23). Therefore, in this study, we selected DSSS as the major scale to acquire patients' information on somatic symptoms.

On account of the importance of somatic symptoms in the mechanism and prognosis of MDD, as well as the reconsideration of existing nosology, we aimed to classify MDD based on the somatic symptoms only. We hypothesized that there could be different trends of somatic symptoms in patients with MDD, and these subtypes would show differences in emotional or other symptoms, as well as treatment responses.

## Methods

### Participants and Procedure

The Algorithm Guided Treatment Strategies for Major Depressive Disorder (AGTs-MDD) study (ClinicalTrials.gov NCT01764867) was a multisite naturalistic cohort, which aimed to compare treatment outcomes between strategies of AGT and Treatment as Usual (TAU) for MDD patients. In brief, the AGTs-MDD cohort screened 1,746 subjects from 8 mental health institutes during 2012 to 2014, in which 964 subjects were diagnosed with MDD according to the criteria of the *Diagnostic and Statistical Manual of Mental Disorders Fourth Edition Text Revision* (DSM-IV-TR). Finally, 845 subjects of Han Chinese were recruited, and they were randomized into AGT (escitalopram or mirtazapine) or TAU group. All procedures complied with the ethical standards of the relevant national and institutional committees on human experimentation and the Helsinki Declaration of 1975, as revised in 2008. The research was approved by the Institutional Review Board of Shanghai Mental Health Center, and all respondents provided written informed consent.

For this study, those with scores of HAMD-17 below 14 or lack of major baseline data were excluded. Besides, we only included subjects that received monotherapy of mirtazapine or escitalopram. Different antidepressants may have different effects on somatic symptoms. For example, escitalopram, as a selective serotonin reuptake inhibitor (SSRI), may cause somatic side effects, such as headache, lower heart rate, and some gastrointestinal symptoms (24). Mirtazapine, as a norepinephrine–serotonin modulator, may cause somatic side effects like some gastrointestinal symptoms and sympathetic activation-related symptoms (25). In this study, the classification of MDD by somatic symptoms was constructed at the baseline, where patients had not accepted any medication. Therefore, the effects on the classification from medications could be neglected. Finally, 395 MDD patients with complete baseline information were selected, in which 311 patients finished a 2-week follow-up, 278 patients finished a 4-week follow-up, 251 patients finished a 6-week follow-up, 199 patients finished an 8-week follow-up, and 178 patients completed a 12-week follow-up.

### Measurements

All patients were assessed using the Depression and Somatic Symptoms Scale (DSSS), the 17-item Hamilton Depression Rating Scale (HAMD-17), the Hamilton Anxiety Rating Scale (HAM-A), the Quality of Life (QOL) Scale, the Global Assessment Function (GAF) Scale, and the International Neuropsychiatric Interview (M.I.N.I.) at baseline. The assessment of DSSS and HAMD-17 scales was completed at every follow-up point. DSSS consists of two subscales, namely, the depression subscale and the somatic subscale (Table 1). Besides, the risk level of suicide was assessed by the total score of M.I.N.I. item C (SUICIDALITY). The total score of HAMD-17 and its reductive rate of score were used to evaluate the treatment responses of subjects.

**Table 1:** Description of symptoms in the questionnaire of DSSS.

**Item**	**Somatic subscale**	**Item**	**Depression subscale**
01	Headache	02	Loss of interest in daily or leisure activities
03	Tightness in the chest	04	Insomnia
05	Muscle tension	06	Irritable mood
07	Back pain	08	Unable to feel happy or decreased ability to fell happy
09	Dizziness	10	Depressed mood or tearful
11	Chest pain	12	Feeling of self-reproach or guilt
13	Neck or shoulder pain	14	Loss of interest in sex
15	Shortness of breath or difficulty breathing	16	Anxious or nervous
17	Soreness in more than half of the body's muscles	18	Unable to concentrate
19	Palpitations or increased heart rate	20	Thoughts of death or suicidal ideas
		21	Fatigue or loss of energy
		22	Decreased appetite or loss of appetite

### Statistical Analysis

Latent profile analysis (LPA) was carried out *via* Mplus 8.3 to explore somatic symptoms-related subtypes of MDD. In the process of LPA, 10 items in the somatic subscale of DSSS were designed as the original items. We fitted one to five latent class models to determine the optimal number of latent classes. A total of six model fit indexes were used to help evaluate the optimal model of LPA: Akaike Information Criterion (AIC), Bayesian Information Criterion (BIC), sample-size adjusted Bayesian Information Criterion (SSABIC), Lo-Mendell-Rubin (LMR), Bootstrapped Likelihood Ratio Test (BLRT), and Entropy. The AIC, BIC, and SSABIC are the information criterion indices used to compare different counterpart models. A lower value indicates a better fitting model. LMR and BLRT are two likelihood ratios used to make a comparison of model fit improvement between models with κ classes and κ-1 classes. A lower and significant *p*-value indicates that the model is superior to the one less class model. Simulation studies have shown that BIC and BLRT are the best indices. Entropy evaluates how well each class could result from LPA. Value exceeding 0.8 is preferred, and approaching 1.0 demonstrates a much better result (26, 27).

Kruskal-Wallis and chi-square (χ^2^) tests were applied for comparing descriptive variables at baseline, including age, sex, body mass index (BMI), medication, depressive subscale items of DSSS, and scores of HAMD-17 and HAM-A, GAF, and QOL scores. The *post-hoc* test, adjusted by the Bonferroni method, was used to conduct a pairwise analysis. Generalized linear mixed models (GLMMs) were adopted to analyze the treatment outcomes of subjects in different subtypes during the 12-week longitudinal follow-ups, and pairwise contrast was performed by the Bonferroni method. The score and reduction rate of HAMD-17 were compared across different LPA subtypes at each follow-up, as well as the scores of depressive and somatic subscales of DSSS. All the statistical analyses were tested bilaterally, with the original significance value set to 0.05.

## Results

### Identification and Description of the Best-Fitting Latent Class

The results of five models (one-class model to five-class model) are presented in Table 2. The 1-class model had the largest AIC, BIC, and ABIC, suggesting the worst model. The 5-class model had the lowest AIC and SSABIC, while with the smallest entropy value. In the 4-class model, BIC, SSABIC, and entropy were smaller than those of the 3-class model, whereas the *P*-value of LMRA was the largest. In view of the LMRA, the 2-class model showed statistical significance; however, the AIC, BIC, and SSABIC were smaller than those of the 3-class model. As shown in Table 2, the 3-class model showed excellent entropy. Therefore, the 3-class model solution pattern yielded optional model values. Ultimately, we decided that the 3-class pattern of the somatic subscale of DSSS was the best-fitting model based on the results from all the six model fit indicators. The correct class assignment probabilities for the 3-class model were excellent, suggesting a good discriminability and a reliable result of LPA with the 3-class model.

**Table 2:** Fit indices of latent profile models of DSSS somatic symptom clusters.

**Model**	**FP**	**AIC**	**BIC**	**SSABIC**	**LMRA (*p*)**	**BLRT (*p*)**	**Entropy**
1	20	10,397.106	10,476.684	10,413.224			
2	31	9,532.997	9,656.342	9,557.979	0.001	<0.001	0.907
3	42	9,146.262	9,313.375	9180,.109	0.183	<0.001	1.000
4	53	9,175.764	9,386.645	9,218.476	0.526	<0.001	0.883
5	64	9,125.107	9,379.756	9,176.683	0.513	>0.05	0.883

*FP, Free parameters; AIC, Akaike's information criterion; BIC, Bayesian information criterion; SSABIC, sample size-adjusted BIC; BLRT, bootstrapped likelihood ratio test; LMRA, Lo-Mendell-Rubin-adjusted likelihood ratio test*.

**Figure 1:**
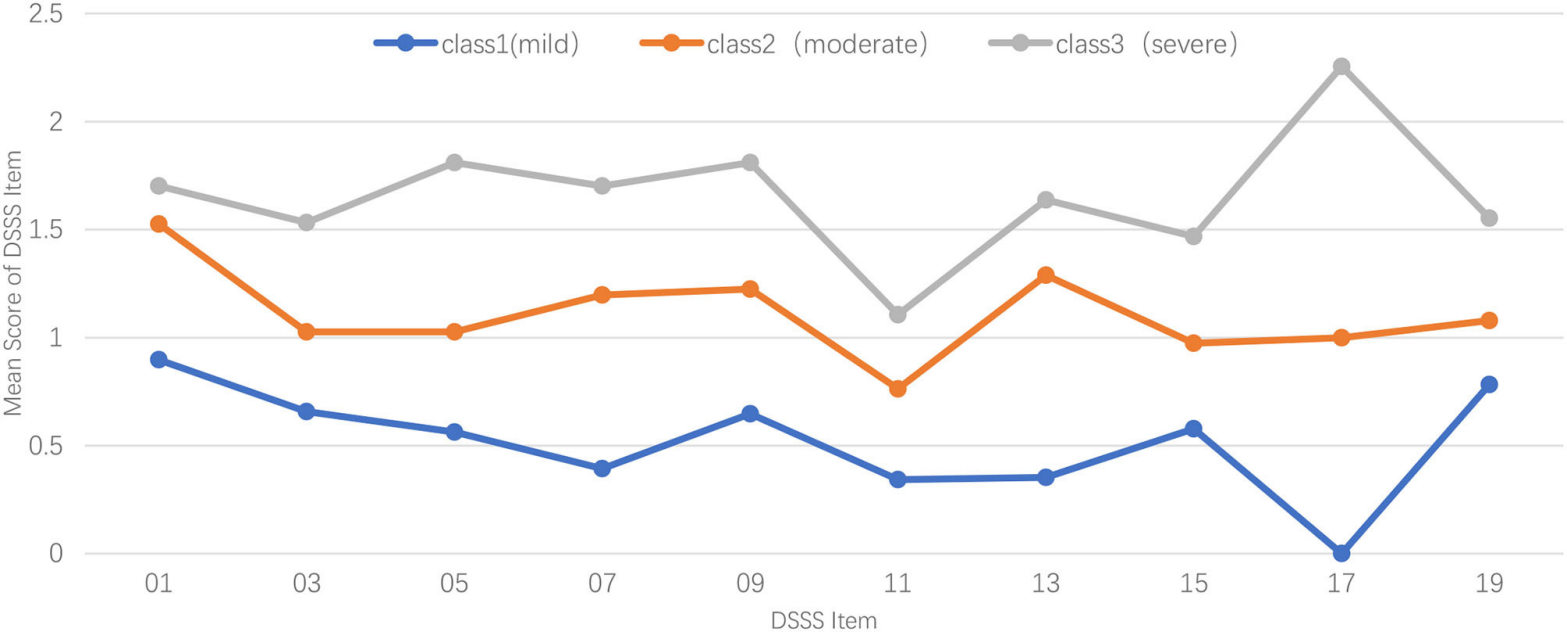
Profiles of latent profile analysis of major depressive disorder by somatic symptoms.

Figure 1 illustrates the profiles of subtypes of somatic symptoms for the 3-class model, in which the Y-axis shows the score of each item, and the X-axis represents different DSSS somatic items that are used for LPA. Participants from class-1 (*n* = 272, 68.9%) were characterized by the lowest scores of somatic items of DSSS, with each item getting the lowest score, especially the symptom of muscle soreness (mea*n* = 0.000). Thus, the 1-class model was labeled as the Mild Group of somatic symptoms. The 2-class model (*n* = 76, 19.2%) showed a similar pattern, with more neck or shoulder pain. Symptoms of muscle tension, dizziness, and body's muscle soreness in 2-class model were moderate between the 1-class model and 3-class model. Given the characteristics of 2-class model, we used the Moderate Group of somatic symptoms to represent it. Participants in the 3-class model (*n* = 47, 11.9%) showed statistically significantly higher somatic symptoms compared with other subtypes, muscle soreness of which was particularly severe (mea*n* = 2.255). Therefore, we named 3-class model as the Severe Group of somatic symptoms.

### Comparison of Clinical Characters of the 3-Class Subtypes at Baseline

As shown in Table 3, age, sex, and BMI of the three groups displayed no statistically significant difference. Besides, the three subgroups showed no difference in medication allocation. We compared the depressive items of DSSS across the three subtypes of MDD, with all items *p-*values <0.01. The 2-class and 3-class models showed higher scores in the symptoms of irritable mood, loss of interest in sex, anxiety or nervousness, unable to concentrate, fatigue or loss of energy, and decreased appetite or loss of appetite. The Severe Group had more problems in losing interest in daily or leisure activities than the other groups. Besides, compared with the Mild Group, the Severe Group showed higher scores in insomnia, unable to feel happy or decreased ability to feel happy, and feeling of self-reproach or guilt. The Moderate Group got the highest score of depressed mood or tearfulness, which was statistically significantly higher than the Mild Group. The total depressive subscale score of the Moderate and Severe Groups was higher than that of the Mild Group. The 3-class subtypes exhibited no differences in the level of sleep problems, suicide risk levels, and GAF (*p* > 0.05). The HAMD-17 scores ascended from 1-class model to 3-class model, and the 1-class model showed the lowest score of HAM-A, which also had better life qualities.

**Table 3:** Comparison of demographic and clinical characteristics across the three subtypes.

**Variable**	**Class 1 mild**	**Class 2 moderate**	**Class 3 severe**	**χ^2^ or *Z***	***p***
	***n =* 272**	***n =* 76**	***n =* 47**		
Age	40.26 (14.23)	38.64 (15.31)	36.68 (12.85)	2.753	0.252
Sex				0.489	0.783
Male	90 (33.1%)	26 (34.2%)	18 (38.3%)		
Female	182 (66.9%)	50 (65.8%)	29 (61.7%)		
BMI	21.66 (3.36)	21.02 (2.78)	21.69 (3.53)	0.943	0.624
Medication				0.131	0.937
Escitalopram	165(61.0%)	46 (61.1%)	27 (58.1%)		
Mirtazapine	107 (39.0%)	30 (38.9%)	20 (41.9%)		
DSSS
02	1.99 (0.81)^a^	1.93 (0.81)^a^	2.36 (0.74)^b^	9.824	0.007
04	1.74 (1.02)^a^	2.04 (0.96)^a, b^	2.23 (0.81)^b^	12.619	0.002
06	1.13 (0.98)^a^	1.66 (0.96)^b^	1.81 (1.17)^b^	26.272	<0.001
08	2.01 (0.77)^a^	2.14 (0.86)^a, b^	2.43 (0.74)^b^	14.276	0.001
10	1.95 (0.79)^a^	2.17 (0.93)^b^	2.15 (0.93)	8.832	0.012
12	1.17 (0.92)^a^	1.41 (1.00)^a, b^	1.62 (0.95)^b^	10.737	0.005
14	0.75 (0.94)^a^	1.26 (0.96)^b^	1.74 (1.09)^b^	45.177	<0.001
16	1.62 (0.86)^a^	2.03 (0.82)^b^	2.17 (0.99)^b^	25.463	<0.001
18	1.45 (0.85)^a^	1.83 (0.93)^b^	2.09 (0.97)^b^	27.046	<0.001
20	0.86 (0.92)^a^	1.20 (0.91)^b^	1.43 (0.97)^b^	20.386	<0.001
21	1.78 (0.78)^a^	2.08 (0.81)^b^	2.30 (0.66)^b^	23.08	<0.001
22	0.97 (0.90)^a^	1.42 (0.91)^b^	1.51 (0.98)^b^	23.236	<0.001
Depressive	17.42 (5.36)^a^	21.17 (5.75)^b^	23.83 (5.75)^b^	57.126	<0.001
Somatic	5.21 (3.94)^a^	11.11 (3.84)^b^	16.57 (5.83)^c^	173.481	<0.001
HAMD-17	20.44 (4.32)^a^	22.56 (5.59)^b^	24.43 (4.11)^c^	32.819	<0.001
HAM-A	16.39 (6.39)^a^	21.99 (6.61)^b^	24.45 (5.92)^b^	78.051	<0.001
GAF	55.89 (9.74)	55.60 (6.56)	51.11 (11.56)	8.314	0.160
QOL	15.48 (2.89)^a^	14.64 (2.77)^b^	13.87 (3.23)^b^	12.949	0.002

*Classes with different alphabets at the top right corner show statistically significant differences in assessment, p <0.05*.

### The Longitudinal Comparison of Treatment Outcome Measures Across Subtypes of MDD

There were dropout rates of 21.3, 29.6, 36.5, 49.6, and 54.9%, respectively, at the 2nd, 4, 6, 8, and 12th weeks. The proportion of patients treated with Escitalopram and Mirtazapine exhibited no difference among the three subgroups both at baseline and at each follow-up point (*p* > 0.05). Scores of HAMD-17 (*F* = 0.2047, *p* = 0.026), somatic (*F* = 23.594, *p* < 0.001), and depressive (*F* = 4.163, *p* < 0.001) items of DSSS among the three groups were statistically different during the follow-up period, while the reduction rate of HAMD-17 score for the three subtypes showed no difference throughout 12 weeks (*F* = 1.303, *p* = 0.238). At the 6th follow-up point, MDD patients with moderate somatic symptoms at baseline (Class 2) had higher scores of HAMD-17 and a lower reduction rate of HAMD-17 than the other two groups (Table 4). Somatic symptoms of the three groups were statistically different until the 12th week, while their depressive symptoms showed similar levels since the 8th week. Besides, from the 4 to 6th weeks, DS scores of the 2-class model were higher than that of the 1-class model and 3-class model (Table 4).

**Table 4:** Longitudinal assessments among the three subtypes by GLMM.

**Week**	**Assessment**	**Class 1**	**Class 2**	**Class 3**	***F***	***p***
2	HAMD-17	13.52(6.09)	15.08(4.66)	13.89(6.17)	1.622	0.198
*n =* 311	Reduction rate	0.34(0.26)	0.31(0.25)	0.40(0.28)	1.502	0.223
	SS	3.83(3.97)^a^	7.08(4.97)^b^	7.56(5.49)^b^	27.660	**<0.001**
	DS	11.77(5.54)^a^	14.00(5.80)^b^	14.00(5.86)^b^	7.177	**0.001**
4	HAMD-17	9.96(5.40)	12.00(4.87)	10.35(6.00)	2.937	0.053
*n =* 278	Reduction rate	0.50(0.26)	0.45(0.25)	0.55(0.27)	2.264	0.104
	SS	2.57(2.89)^a^	5.09(4.71)^b^	5.79(5.44)^b^	16.870	**<0.001**
	DS	8.68(5.13)^a^	11.43(5.79)^b^	10.18(5.49)^a, b^	6.074	**0.002**
6	HAMD-17	8.20(5.32)^a^	11.29(5.62)^b^	8.45(6.52)^a^	5.973	**0.003**
*n =* 251	Reduction rate	0.59(0.27)^a^	0.47(0.28)^b^	0.63(0.30)^a^	4.576	**0.010**
	SS	2.13(2.93)^a^	4.63(4.68)^b^	4.87(5.90)^b^	12.634	**<0.001**
	DS	7.44(5.13)^a^	9.56(6.10)^b^	8.27(5.88)^a, b^	3.018	**0.049**
8	HAMD-17	5.62(4.21)	7.29(4.96)	7.71(5.58)	2.650	0.071
*n =* 199	Reduction rate	0.71(0.22)	0.67(0.22)	0.67(0.25)	0.691	0.501
	SS	1.41(2.34)^a^	2.69(3.22)^b^	3.88(4.47)^b^	5.709	**0.003**
	DS	5.33(4.34)	5.89(5.06)	7.42(5.45)	1.269	0.282
12	HAMD-17	4.76(4.32)	6.97(5.42)	5.45(4.87)	2.954	0.052
*n =* 178	Reduction rate	0.75(0.24)	0.67(0.27)	0.78(0.21)	2.669	0.070
	SS	1.11(1.69)	2.38(2.85)	2.76(3.40)	2.955	0.052
	DS	3.97(3.87)	5.56(5.12)	5.75(4.59)	1.422	0.241

*Bold values indicate p <0.05. SS, somatic subscale of DSSS; DS, depressive subscale of DSSS. Classes with different alphabets at the top right corner show statistically significant differences in assessment, p <0.05*.

## Discussion

The LPA found three subtypes of MDD, namely, depression with mild somatic symptoms, depression with moderate somatic symptoms, and depression with severe somatic symptoms. The 3-class model showed excellent membership classification with an entropy score of 1.000. This finding indicated that the severity of somatic symptoms might be a basis for MDD classification. Comparisons among the three groups showed that MDD patients with severer somatic symptoms had more problems with depressive symptoms and anxiety. Notably, cohort comparison among the three subtypes found that it was the Moderate Group rather than the Severe Group that had the worst remission. The Moderate Group displayed a higher HAMD-17 score at the 6th week and severer depressive symptoms both at the 4 and 6th weeks follow-up points.

Due to the population heterogeneity of MDD, researchers have been motivated to identify homogeneous clinically useful subtypes of MDD for purchasing a better prognosis and understanding of this disease. Although biological parameters seem to be more objective and have less bias, symptoms are what psychiatrists directly assess in clinical practice. Early symptom-based subtyping studies have labeled and validated the “melancholic” and the “non-melancholic” subtypes of MDD (28, 29). Later on, researchers found three stable subtypes of MDD, including the moderate subtype and the severe subtype with hypersomnia, increased appetite, and weight, and the severe subtype with diurnal variation, insomnia, early morning awakening, and decreased appetite and weight (30, 31). A recent study used both depressive and anxiety symptomatic items to cluster MDD (32). Besides, some studies have tried to combine clinical questionnaire scores and biological parameters, such as plasma indexes and gene expressions, to cluster MDD subjects at a high dimension (33, 34). Unlike previous studies, this study focused only on the somatic symptoms of MDD subtyping, which displayed a new aspect for understanding MDD.

This study provided evidence of the comorbidity of somatic and depressive symptoms in patients with MDD. Those with more severe somatic symptoms also had more serious problems with depressive symptoms. The presence of major depressive episode has been proved to be strongly correlated with the loss of interest (35), which is also a distinguishing feature for the subgroup with severe somatic symptoms. The Severe Group had more complaints of insomnia than the Mild Group, while previous research reported that in both adolescents and adults, subjects with insomnia scored higher on somatic symptom measurements than non-insomnia ones (36). Guidi et al. found two clusters of MDD, including depressed somatizers and irritable/anxious depression (37). This study further proved that patients with milder somatic symptoms also suffered less from irritable mood. Previously, a study demonstrated that somatic symptoms of depression had an influence on the ability of being happy (38), which was reconfirmed in this study that the Severe Group exhibited more problems in feeling happy. What surprised us is that it was the Moderate Group rather than the Severe Group that showed heavier depressed mood, which suggested that the more severe somatic symptoms might cover the symptoms of emotion to some degree. Subjects in the Severe Group were found to be more likely to feel guilt or self-reproach. It has been reported that guilt may function as a mediator between childhood trauma and adult somatic symptoms (39), suggesting that patients with more severe somatic distress might have experienced something unusual in their childhood, which should not be neglected by psychiatrists in clinical practice. We found that the interest in sex also differed among the three groups. However, a previous report declared that the severity of sexual dysfunction was uncorrelated with the somatic dimension (40). The reason might be that the loss of interest in sex is more related to the lack of pleasure but not sexual functions. The three groups were found to have ascending levels of anxiety, which was consistent with previous studies (41). The Moderate and Severe groups showed heavier concentration problems; however, there existed an argument on whether to divide concentration into the cognitive or somatic cluster (42). A recent study reported that heavier burdens of somatic symptoms were associated with the risk of suicide (43), which has been further proved in this study. As indicated by previous studies (44), subjects with different degrees of somatic symptoms also differ in the symptom of energy and appetite loss.

The outcomes of the three subtypes were compared in a clinical trial. The STAR^*^D Study reported that patients with somatic depression exhibited low remission rates in response to citalopram (45). Mirtazapine was reported to be an effective and safe antidepressant for depressive patients with somatic symptoms (46). Although mirtazapine shows stronger effects for somatic symptoms, in this study, medication allocation of the three subgroups displayed no statistical difference both at baseline and at each follow-up point. Therefore, the impact of drugs on curative effects among groups could be excluded. After the 6-week medication, the group with moderate somatic symptoms exhibited the highest score of HAMD-17, as well as the lowest reductive rate. A previous study reported that patients with no/mild somatic symptoms (84.1%) achieved the highest proportion of remission than those with moderate or severe somatic symptoms (72.0 and 55.3%) (47). In this study, we found that patients with moderate somatic symptoms actually had the worst remission and severe depressive symptoms. It provides a warning for psychiatrists that more attention is needed for MDD patients with moderate somatic symptoms (somatic subscale of DSSS scores from 11.11 to 16.57) in clinical work.

Previous evidence has proved that there is an association between neurovegetative symptoms of depression and inflammation (48). Besides, in patients with MDD, inflammation-related proteins, such as C-reactive protein (CRP) and interleukin-6 (IL-6), were positively connected to somatic symptoms (49). In this study, patients with moderate to severe somatic symptoms tended to have problems with fatigue or loss of energy and decreased appetite or loss of appetite. Alessandro et al. observed a strong association between altered appetite/eating symptoms with CRP and white blood cell count (WBC), as well as between tiredness/low energy with granulocyte-to-lymphocyte ratio (GLR) (50). Therefore, there could be potential inflammation-related changes among the three groups, which might imply clues for the mechanism under the classification and their prognosis. In future studies, inflammatory proteins of patients with different levels of somatic symptoms are supposed to be examined for a better understanding of MDD and seeking for more specific treatment target.

## Limitations

There were several main limitations of this study to be noted. First, there was a relatively high dropout rate, especially for the longer treatment follow-ups. A high dropout rate may be due to that patients were required to stick at least 6 weeks to monotherapy, and those who could not receive ideal remission might choose another drug and then drop out of the study. Second, this study only focused on the treatment outcome of the acute phase of 12 weeks, not on a long-term outcome, and further studies are required to explore a longer follow-up period of MDD patients with different levels of somatic symptoms. Third, this study failed to make a validation of the 3-class subtype model with external data, such as patients from other hospitals. Finally, the number of participants varies significantly among the three subgroups, which, to some degree, is due to the characteristics of the subtypes, and it might also result from the small sample size. Thus, a future study with a larger sample size is needed to assure the accuracy of the subtype model.

## Conclusion

This study focused only on the somatic symptoms of MDD subtyping, which displayed a new aspect for understanding MDD. The results of this study identified three subtypes of MDD by their somatic symptoms: MDD patients with mild somatic symptoms, MDD patients with moderate somatic symptoms, and MDD patients with severe somatic symptoms. We found that patients with more severe somatic symptoms showed more problems with depressive symptoms and anxiety. The cohort comparison of the three subtypes found that it was the Moderate Group rather than the Severe Group that had the worst remission. The Moderate Group had higher HAMD-17 scores at the 6th week. It provides a warning that in clinical practice, somatic symptoms should be emphasized both to patients and doctors, and more attention is needed for MDD patients with moderate somatic symptoms, to whom a longer medication is supposed to be applied. Since few antidepressants have been studied for targeting somatic symptoms, future studies should pay more attention to somatic symptoms in MDD patients, and figure out more evidence of accurate pharmacotherapy for MDD with somatic symptoms.

## Data Availability Statement

The data that support the findings of this study are available from SMHC. Restrictions apply to the availability of these data, which were used under license for this study.

## Ethics Statement

The studies involving human participants were reviewed and approved by Shanghai Mental Health Center Institution Review Board Office. The patients/participants provided their written informed consent to participate in this study.

## Author Contributions

XW: data analysis, methodology, writing original draft, reviewing, and editing. YZ, YW, DP, and JC: conceptualization, methodology, reviewing, and editing. ZW: conceptualization, methodology, data analysis, reviewing, and editing. JH: conceptualization, methodology, and project administration. LC, YW, YS, and HL: conceptualization, reviewing, and editing. MF, ZY, ZW, and FW: data curation, investigation, and methodology. YF: conceptualization, funding acquisition, investigation, methodology, project administration, resources, supervision, reviewing, and editing. All authors contributed to the article and approved the submitted version.

## Funding

This study was supported by the National Key R&D Program of China (2016YFC1307100 and 2012BAI01B04), the National Natural Science Foundation of China (81930033 and 81771465), the Cross-Disciplinary and Translational Medical Research of Shanghai Jiao Tong University (ZH2018ZDA29), the Sanming Project of Medicine in Shenzhen (SZSM201612006), the Shanghai Municipal Science and Technology Major Project (2018SHZDZX05), and the Innovative Research Team of High-level Local Universities in Shanghai.

## Conflict of Interest

The authors declare that the research was conducted in the absence of any commercial or financial relationships that could be construed as a potential conflict of interest.

## Publisher's Note

All claims expressed in this article are solely those of the authors and do not necessarily represent those of their affiliated organizations, or those of the publisher, the editors and the reviewers. Any product that may be evaluated in this article, or claim that may be made by its manufacturer, is not guaranteed or endorsed by the publisher.
